# Overall side effect assessment of oxaliplatin toxicity in rectal cancer patients in NRG oncology/NSABP R04

**DOI:** 10.1007/s11136-024-03746-5

**Published:** 2024-07-30

**Authors:** John Devin Peipert, Jessica Roydhouse, Mourad Tighiouart, Norah Lynn Henry, Sungjin Kim, Ron D. Hays, Andre Rogatko, Greg Yothers, Patricia A. Ganz

**Affiliations:** 1 Department of Medical Social Sciences, Northwestern University Feinberg School of Medicine, 625 Michigan Ave, 22nd Floor, Chicago, IL 60611 USA; 2 Menzies Institute for Medical Research, University of Tasmania, Hobart, Australia; 3 Samuel Oschin Comprehensive Cancer Institute, Cedars-Sinai Medical Center, Los Angeles, CA USA; 4 University of Michigan Medical School, Ann Arbor, MI USA; 5 Division of General Internal Medicine and Health Services Research, Department of Medicine, David Geffen School of Medicine at University of California, Los Angeles, CA USA; 6 University of Pittsburgh and NRG Oncology, Pittsburgh, PA USA; 7 Department of Health Policy and Management, UCLA Fielding School of Public Health, Los Angeles, CA USA; 8 Department of Medicine (Hematology/Oncology), David Geffen School of Medicine at University of California, Los Angeles, CA USA

**Keywords:** Cancer, Treatment tolerability, FACT GP5, Clinical trials

## Abstract

**Purpose:**

Regulatory guidance suggests capturing patient-reported overall side effect impact in cancer trials. We examined whether the Functional Assessment of Cancer Therapy (FACT) GP5 item (“I am bothered by side effects of treatment”) post-neoadjuvant chemotherapy/radiotherapy differed between oxaliplatin vs. non- oxaliplatin arms in the National Surgical Adjuvant Breast and Bowel Project (NSABP) R-04 trial of stage II–III rectal cancer patients.

**Methods:**

The R-04 neoadjuvant trial compared local-regional tumor control between patients randomized to receive 5-fluorouracil or capecitabine with radiation, with or without oxaliplatin (4 treatment arms). Participants completed surveys at baseline and immediately after chemoradiotherapy. GP5 has a 5-point response scale: “Not at all” (0), “A little bit” (1), “Somewhat” (2), “Quite a bit” (3), and “Very much” (4). Logistic regression compared the odds of reporting moderate-high side effect impact (GP5 2–4) between patients receiving oxaliplatin or not after chemoradiotherapy, controlling for relevant patient characteristics. We examined associations between GP5 and other patient-reported outcomes reflecting side effects.

**Results:**

Analyses were performed among 1132 study participants. Participants receiving oxaliplatin were 1.58 times (95% CI: 1.22–2.05) more likely to report moderate-high side effect bother at post-chemotherapy/radiation. In both arms, worse overall side effect impact was associated with patient-reported diarrhea, nausea, vomiting, and peripheral sensory neuropathy (*p* < 0.01 for all).

**Conclusion:**

This secondary analysis of R-04 found that GP5 distinguished between patients receiving oxaliplatin or not as part of their post-neoadjuvant chemoradiotherapy, adding patient-centric evidence on the reduced tolerability of oxaliplatin and demonstrating that GP5 is sensitive to known toxicity differences between treatments.

**ClinicalTrials.gov:**

NCT00058474.

**Supplementary Information:**

The online version contains supplementary material available at 10.1007/s11136-024-03746-5.

## Introduction

Oncology treatments often carry a severe side effect profile, which can impact health-related quality of life (HRQoL) and willingness to remain on therapy. Recognition of this impact has spurred regulators, patients, and advocacy groups to encourage the collection of information about how patients feel and function during therapy [1, 2]. One aspect of tolerability covered in recent regulatory guidance is the overall side effect impact. The Functional Assessment of Cancer Therapy (FACT) measurement system, which has been widely used in cancer trials, includes the GP5 single item of overall side effect impact (“I am bothered by side effects of treatment”) [3]. Understanding if this item can differentiate between treatment arms of varying side effects is essential for determining the usefulness and interpretation of this item in informing treatment decision-making in oncology.

There are estimated to be over 46,000 new cases of rectal cancer diagnosed in the United States in 2023 (approximately 60% men, 40% women) [4]. For patients with stage II and III disease with resectable rectal cancer, pre-operative neoadjuvant chemotherapy combined with radiotherapy has been a standard of care for several decades. The National Surgical Adjuvant Breast and Bowel Project (NSABP) has serially tested different combinations of these therapeutic approaches over the past two decades [5, 6]. The NSABP R-04 trial (NCT00058474) was conducted between 2004 and 2010 and was originally designed to compare the effects of the chemotherapy regimens 5-Fluorouracil (5-FU) and capecitabine, both in combination with radiotherapy, on local-regional tumor control, disease-free survival, and overall survival [7]. A protocol amendment in 2005 added oxaliplatin to each original treatment, creating four total arms: (1) 5-FU + radiotherapy alone; (2) oxaliplatin + 5-FU + radiotherapy; (3) capecitabine + radiotherapy alone; (4) oxaliplatin + capecitabine + radiotherapy. Though no difference between treatment arms was observed on the primary endpoint of local-regional recurrence or secondary endpoints in R-04 [7], patients in the oxaliplatin treatment arms had higher proportions of grade 3–5 adverse events in comparison to the non-oxaliplatin arms [7]. As a result of greater toxicity and no benefit in efficacy, oxaliplatin has not been adopted as part of the treatment strategy for rectal cancer.

The NSABP R-04 trial included a patient-reported outcome (PRO) sub-study that included a cancer-specific instrument, a symptom checklist that focused on toxicity related to 5-FU and capecitabine, and neurotoxicity. The detailed results of that sub-study documented expected toxicities of the regimens, including greater neurotoxicity and diarrhea in the oxaliplatin containing arms of the trial [8, 9]. In addition, as part of a secondary analysis focused on developing new methods of evaluation of cancer treatment tolerability, Gresham et al. [10]. applied the Toxicity Index (TI) method to examine Common Terminology Criteria for Adverse Events (CTCAE) toxicities reported at the end of chemoradiation in the R-04 trial. They found the greatest number of toxicities in the oxaliplatin arms and that women experienced significantly greater treatment toxicity across the treatment groups [10].

There has been growing regulatory and clinical interest in more direct approaches to capture treatment tolerability from the patient’s perspective [1, 11–13], in part due to the recognition that clinician reports of symptomatic side effects collected as part of adverse event assessment may underestimate negative effects on patients [14]. Since the GP5 item captures overall treatment tolerability without reference to specific symptomatic side effects, it may help characterize the tolerability of different treatments within a clinical trial and the cumulative effects of multiple toxicities [2, 11]. The GP5 item was administered in NSABP R-04, but not examined in the previous PRO analyses. Given the higher rates of both gastrointestinal toxicity and neurotoxicity among patients receiving oxaliplatin in R-04 [7], there is particular interest in whether the overall side effect experience was worse when this drug was combined with either 5-FU or capecitabine, compared to each of the latter used alone. Therefore, the aims of this study were to test whether the distribution of responses to the GP5 item at post-neoadjuvant chemotherapy/radiotherapy differed significantly between treatment arms, and to determine whether post-neoadjuvant chemotherapy/radiotherapy GP5 is associated with other PRO measures reflecting symptomatic side effects of treatment.

## Methods

### NSABP R04 trial

NSABP R-04 (NCT00058474) was a multi-center study conducted largely in the United States that, after an amendment adding oxaliplatin, randomized participants 1:1:1:1 to receive one of the four treatment arms: (1) 5-FU + radiotherapy alone; (2) oxaliplatin + 5-FU + radiotherapy; (3) capecitabine + radiotherapy alone; (4) oxaliplatin + capecitabine + radiotherapy. All participants received radiotherapy at 180 cGy per day, five days per week. Participants randomized to receive 5-FU received this treatment by continuous intravenous infusion at 225 mg/m2 per day 5 days/week (reduced from 7 days a week at the time of the amendment adding oxaliplatin) from the start of radiation therapy to the last dose of radiation therapy. Patients randomized to receive capecitabine received this treatment at 825 mg/m2 po bid 5 days a week (also reduced from 7 days a week at the time of the amendment adding oxaliplatin) from the start to the end of radiation therapy. Oxaliplatin was administered at 50 mg/m2 IV weekly during radiation therapy for 5 weeks. Patients were eligible to participate in R-04 if they were *≥* 18 years old and had an Eastern Cooperative Oncology Group (ECOG) performance score of 0–1. Prior to randomization, a clinician determined whether sphincter-sparing surgery was feasible. Detailed eligibility and screening information has been published elsewhere [7].

Participants speaking English, French, or Spanish were invited to participate in a PRO sub-study. Those agreeing were administered a PRO questionnaire prior to randomization (baseline) and after completion of their randomized radiation/chemotherapy regimen (post-neoadjuvant therapy and prior to surgery). They also completed the questionnaire 12 months after surgery. In cases where radiation/chemotherapy was delayed, these measures were completed after radiotherapy to capture the patient’s assessment at the end of therapy, whenever that occurred. The PRO questionnaire was collected on schedule after discontinuation of treatment for reasons other than recurrence or a second primary cancer diagnosis. If the PRO questionnaire was not completed at an assessment time point, the clinical study coordinator was required to complete a form that provided the reason for missing data that was sent to the statistical coordinating center. All PROs were self-administered as paper forms.

### PRO questionnaire

Several PRO instruments were included in the questionnaire. The focal measure for the current study is the single item GP5, “I am bothered by side effects of treatment,” which has response options of “Not at all” (0), “A little bit” (1), “Somewhat” (2), “Quite a bit” (3), and “Very much” (4). The GP5 item is included in the FACT-Colorectal (FACT-C) [15], which contains the FACT – General (FACT-G), including its subscales for Physical Well-Being, Social/Family Well-Being, Emotional Well-Being, Functional Well-Being, and Colorectal Cancer. For each FACT-C scale, higher scores indicate better HRQoL or lower symptom bother, though some single items have response options with higher scores indicating worse symptoms; these are reversed prior to scoring scales. The NSABP Symptom Checklist (SCL-17) includes an 11-item 5-FU–specific symptom scale and 6 single items capturing impacts of colorectal cancer and treatment [16]. The SCL-17 score is the average of 17 items with a 0 to 100 range and higher scores indicating worse HRQoL or higher symptom burden. The EORTC-QLQ-CR38 covers functional impact and symptoms related to colorectal cancer with multiple multi-item scales (e.g., Chemotherapy Side Effects) and single items [17]. All EORTC-QLQ-CR38 scales and single-item scores were linearly transformed to a 0–100 scale with higher scores indicating worse HRQoL or higher symptom bother. Finally, two versions of the FACT-Neurotoxicity (NTX) measures were administered, the NTX13 and the NTX4, and each covers multiple manifestations of neurotoxicity related to cancer treatments, such as peripheral neuropathy [18]. Scores are generated using a similar procedure as the FACT-C, and the NTX13 has a 0 to 52 range while the NTX4 has a 0 to 16 range, with higher scores representing worse neurotoxicity. (This direction of scoring is non-standard for FACT- NTX scales.)

### Statistical analyses

The primary hypothesis tested in our analyses was whether the GP5 detected worse overall side effect impact in treatment arms containing oxaliplatin than in arms without oxaliplatin immediately after chemoradiotherapy. After testing this hypothesis, we examined associations between GP5 response and PRO scale scores and single items measuring specific symptomatic side effects to explore which treatment toxicities contribute most to worse overall side effect impact. We hypothesized that scales and items representing gastrointestinal issues and neurotoxicity would be most likely to be associated with GP5.

Participants’ demographic and clinical characteristics were summarized using frequencies and proportions or medians and interquartile ranges (IQR). These included age at study start in years, gender (men, women), race/ethnicity (Hispanic, non-Hispanic Black, non-Hispanic White, non-Hispanic other/unknown race), body mass index (BMI; underweight = BMI < 18.5; Normal weight = 18.5 < = BMI < 25; overweight = 25 < = BMI < 30; obesity = BMI > = 30), Karnofsky performance status, clinical stage, and whether there was surgical intent to save the sphincter. We examined associations between each of the patient characteristics and GP5 response at post- radiation/chemotherapy with Kruskal-Wallis tests or Spearman rank correlation as appropriate.

To test our primary hypothesis, bivariate and multivariable logistic regression models were used to estimate the odds of reporting moderate-to-high bother on GP5 (defined as “Somewhat”/“Quite a bit”/“Very much”) vs. low bother (“Not at all”/“A little bit”) [3] at post-radiation/chemotherapy as a function of treatment arm of randomization (oxaliplatin vs. not) with and without adjustment for baseline GP5 and participant characteristics listed above, which were selected a priori based on clinical expertise and informed by previously-demonstrated associations between participant characteristics and HRQoL in the R-04 trial [8]. All covariates were entered simultaneously in the multivariable model. Model diagnostics were performed to ensure that the logistic model was appropriate. In multivariable analysis, multicollinearity was assessed by tolerance and variance inflation factor and an interaction effect between the oxaliplatin treatment and gender was examined. Estimates are presented as odds ratios and corresponding 95% confidence intervals. All odds ratios in the multivariable model account for the effects of all other covariates.

To examine our secondary hypotheses, probabilistic index models [19–23] were used to examine the associations between each of the PRO scales representing key symptomatic side effects and GP5 response post-radiation/chemotherapy (ordinal variable, 0=“Not at all”, 1=“A little bit, 2=“Somewhat”, 4=“Quite a bit”, 5=“Very much”) stratified by treatment with and without oxaliplatin. PRO scale scores included FACT-C subscale scores and selected individual items, EORTC-CR38 subscale scores and selected individual items, selected SCL-17 items, and FACT-NTX scores, with a specific focus on gastro-intestinal impacts and neurotoxicity. These models estimate the probability that a GP5 response value for one group is greater (indicating more severe or worse bother) than or equal to the value for another group along with a Wald-type 95% confidence interval. P-values were calculated using Wald-statistics and adjusted for multiple tests using the Holm procedure [24]. A probability of 0.50 would indicate no difference in GP5 response between participants with a 1-unit increase in the PRO scale score (worsening or improvement varies by scale) and those who did not. A probability of > 0.50 indicates that a 1-unit increase in the PRO scale score is associated with worse or more severe side effect bother.

Analyses were performed using R package version 4.0.5 [25] with two-sided tests at the significance level of 0.05.

## Results

In total, the study enrolled and randomized 1608 patients. Of these, 1373 patients had baseline PRO data and follow-up PRO data. Additionally, 1132 of the 1373 were randomized to one of the 4-arm study groups after the trial was amended to add oxaliplatin; this was the analysis sample for the current study. A CONSORT (Consolidated Standards of Reporting Trials) diagram for patient selection is shown in Supplementary Fig. 1. Table 1 shows the distribution of patient characteristics among the analytic sample. The distribution of patients randomized to each treatment arm was: 5-FU alone, *n* = 277 (24.4%); 5-FU + oxaliplatin, *n* = 286 (25.3%); capecitabine alone, *n* = 283 (25.0%); capecitabine + oxaliplatin, *n* = 286 (25.3%). Collapsing these categories, 572 participants (50.5%) were randomized to receive oxaliplatin. On average, participants were 57.2 years old, and the largest proportions were men (*n* = 777, 68.6%), Non-Hispanic White (*n* = 965, 85.3%), obese (*n* = 414, 36.6%), had Karnofsky performance status 90–100 (958, 84.6%), clinical stage III (*n* = 698, 61.7), and with intent for sphincter saving (*n* = 839, 74.1%).

**Table 1:** Baseline patient characteristics (N = 1132)

Variable	n (%) or median (IQR)
Treatment	
5-FU (4 Arm)	277 (24.4%)
5-FU + Oxa (4 Arm)	286 (25.3%)
Cape (4 Arm)	283 (25.0%)
Cape + Oxa (4 Arm)	286 (25.3%)
Oxaliplatin at post-randomization	572 (50.5%)
Age, years	57.5 (11.2)
Gender	
Male	777 (68.6%)
Female	355 (31.4%)
Race/Ethnicity	
Non-Hispanic Black	49 (4.3%)
Hispanic	61 (5.4%)
Non-Hispanic Other/Unknown	57 (5.0%)
Non-Hispanic White	965 (85.3%)
BMI (kg/m^2^)^a^	
Underweight BMI < 18.5	13 (1.2%)
Normal weight 18.5 < = BMI < 25	300 (26.5%)
Overweight 25 < = BMI < 30	405 (35.8%)
Obesity BMI > = 30	414 (36.6%)
Karnofsky Performance Status	
90–100	958 (84.6%)
50–80	174 (15.4%)
Clinical Stage	
II	433 (38.3)
III	698 (61.7)
Intent to save sphincter	839 (74.1%)

^a^One patient’s height was imputed using age and gender

**Fig. 1:**
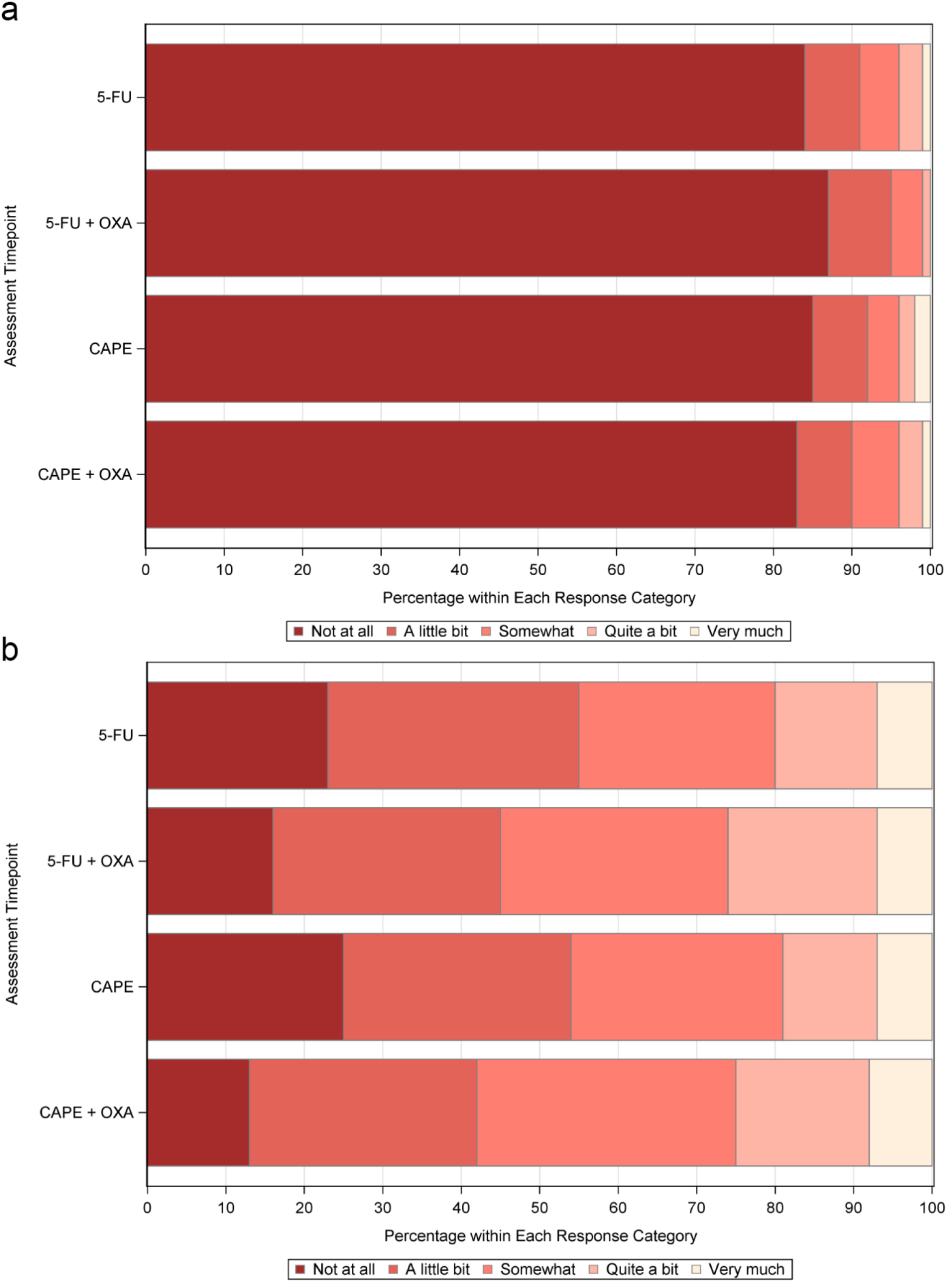
Distribution of GP5 Responses by Treatment Arm at Baseline and Post-Chemo/Radiation Panel (**A**) Baseline. Panel (**B**) Post-Chemotherapy/Radiation

At baseline, 1013 of 1132 (89.5%) participants and at post-chemoradiation 1082 of 1087 (99.5%) had GP5 data. Figure 1 shows the frequency distribution of GP5 responses at baseline (Panel A) and post-chemotherapy/radiation (Panel B) stratified by treatment arm. At baseline, the largest proportions (> 80%) in each arm reported being “Not at all” bothered by side effects. At post-chemotherapy/radiation, these distributions changed substantially, with participants receiving oxaliplatin having larger proportions of patients reporting moderate to high bother on GP5 (“Somewhat”/ “Quite a bit”/“Very much”) in comparison to the two arms not containing oxaliplatin. Table 2 shows associations between the treatment arm of randomization and participant characteristics and GP5 response at post-chemotherapy/radiation. Treatment arm was significantly associated with GP5 response (*p* < 0.001), with larger proportions of the oxaliplatin-containing arms responding “Somewhat” or “Quite a bit.” Women were significantly more likely to respond “Quite a bit” or “Very much” than men (*p* < 0.001). Participants with Karnofsky performance status of 50–80 were more likely to respond “Very much” than those with 90–100 (*p* < 0.001).

**Table 2:** Associations between patient characteristics and GP5 response at post-radiation/chemotherapy

Variable	GP5 Response at Post-Radiation/Chemotherapy					
	Not at all (N = 207)	A little bit (N = 323)	Somewhat (N = 309)	Quite a bit (N = 164)	Very much (N = 79)	P-value
**Treatment**						< 0.001
5-FU (4 Arm)	61 (23.1%)	84 (31.8%)	67 (25.4%)	34 (12.9%)	18 (6.8%)	
5-FU + Oxa (4 Arm)	43 (16.0%)	79 (29.5%)	76 (28.4%)	51 (19.0%)	19 (7.1%)	
Cape (4 Arm)	67 (24.8%)	80 (29.6%)	72 (26.7%)	32 (11.9%)	19 (7.0%)	
Cape + Oxa (4 Arm)	36 (12.9%)	80 (28.6%)	94 (33.6%)	47 (16.8%)	23 (8.2%)	
**Treatment**						< 0.001
Oxaliplatin	79 (14.42)	159 (29.01)	170 (31.02)	98 (17.88)	42 (7.66)	
No Oxaliplatin	128 (23.97)	164 (30.71)	139 (26.03)	66 (12.36)	37 (6.93)	
**Age (Years)**,** Median (IQR)**	59.1 (16.0)	57.7 (15.7)	55.52 (15.3)	57.2 (15.8)	59.5 (19.4)	0.076
**Gender**						< 0.001
Male	150 (20.2%)	236 (31.9%)	220 (29.7%)	99 (13.4%)	36 (4.9%)	
Female	57 (16.7%)	87 (25.5%)	89 (26.1%)	65 (19.1%)	43 (12.6%)	
**Race/Ethnicity**						0.128
Non-Hispanic Black	8 (16.33)	18 (36.7%)	17 (34.7%)	2 (4.1%)	4 (8.2%)	
Hispanic	13 (22.0%)	16 (27.1%)	12 (20.3%)	10 (17.0%)	8 (13.6%)	
Non-Hispanic Other/ Unknown	6 (10.7%)	13 (23.2%)	20 (35.7%)	13 (23.2%)	4 (7.1%)	
Non-Hispanic White	180 (19.6%)	276 (30.1%)	260 (28.3%)	139 (15.1%`)	63 (6.9%)	
**BMI (kg/m** ^**2**^ **)**						0.07
Underweight BMI < 18.5	1 (8.3%)	3 (25.0%)	5 (41.7%)	1 (8.3%)	2 (16.7%)	
Normal weight 18.5 < = BMI < 25	39 (13.5%)	88 (30.34%)	94 (32.4%)	47 (16.2%)	22 (7.6%)	
Overweight 25 < = BMI < 30	75 (19.7%)	128 (33.6%)	100 (26.3%)	50 (13.1%)	28 (7.4%)	
Obesity BMI > = 30	92 (23.1%)	104 (26.1%)	110 (27.6%)	66 (16.5%)	27 (6.8%)	
**Karnofsky Performance Status**						< 0.001
Karnofsky 90–100	178 (19.5%)	279 (30.5%)	255 (27.9%)	141 (15.4%)	62 (6.8%)	
Karnofsky 50–80	29 (17.4%)	44 (26.4%)	54 (32.3%)	23 (13.8%)	17 (10.2%)	
**Clinical Stage**						0.762
II	133 (19.9%)	197 (29.5%)	182 (27.3%)	106 (15.9%)	49 (7.4%)	
III	73 (17.6%)	126 (30.4%)	127 (30.7%)	58 (14.0%)	30 (7.3%)	
**Intent to save sphincter**						0.71
Yes	60 (21.1%)	81 (28.4%)	79 (27.7%)	44 (15.4%)	21 (7.4%)	
No	147 (18.4%)	242 (30.4%)	230 (28.9%)	120 (15.1%)	58 (7.3%)	

Data are presented as number of patients (row %) or median (IQR, interquartile range)

P-value is calculated by Kruskal-Wallis test or Spearman rank correlation as appropriate

Table 3 shows the results of logistic regression analyses examining associations of oxaliplatin treatment, baseline GP5, and participant characteristics with responding with moderate-high bother on GP5 (“Somewhat”/“Quite a bit” /“Very much” vs. “A little bit”/“Not at all”) at post-chemotherapy/radiation. The largest effect was for baseline GP5; participants reporting moderate-high bother on GP5 at baseline were 2.3 times (95% CI: 1.35–3.93) more likely to give this response at post-chemotherapy/radiation after adjusting for patient characteristics in the multivariable model. In the multivariable model, participants randomized to receive oxaliplatin were 1.58 times (95% CI: 1.22–2.05) more likely to report moderate-high side effect bother at post-chemotherapy/radiation than those not receiving oxaliplatin. Finally, in the multivariable model, women were 1.53 times (95% CI: 1.15–2.04) more likely to report moderate-high side effect bother at post-chemotherapy/radiation than men. We note that the treatment by gender interaction term was not statistically significant (p-value = 0.956; not shown in table).

**Table 3:** Bivariate and multivariable analyses of GP5 at post-radiation/chemotherapy with oxaliplatin (OXA) receipt and baseline patient demographic and clinical characteristics

Independent Variable	Bivariate		Multivariable^a^	
	Odds Ratio (95% CI)	P-value	Odds Ratio (95% CI)	P-value
GP5 at baseline = Somewhat/Quite a bit/Very much (reference = Not at all/A little bit)	2.55 (1.53–4.26)	< 0.001	2.30 (1.35–3.93)	0.002
OXA (reference = no OXA at randomization)	1.57 (1.24-2.00)	< 0.001	1.58 (1.22–2.05)	< 0.001
Age at entry (years) b	0.99 (0.98-1.00)	0.078	0.99 (0.98-1.00)	0.167
Female gender (reference = male)	1.49 (1.15–1.93)	0.003	1.53 (1.15–2.04)	0.003
Race/ethnicity (reference = Non-Hispanic White)				
Non-Hispanic Black	0.87 (0.49–1.55)	0.644	0.88 (0.47–1.63)	0.675
Hispanic	1.02 (0.60–1.73)	0.938	0.83 (0.46–1.49)	0.54
Non-Hispanic Other/Unknown	1.92 (1.09–3.39)	0.024	1.48 (0.78–2.79)	0.228
BMI (kg/m2; reference = obesity BMI > = 30)				
Underweight BMI < 18.5	1.93 (0.57–6.51)	0.289	1.21 (0.31–4.71)	0.788
Normal weight 18.5 < = BMI < 25	1.24 (0.91–1.68)	0.167	1.26 (0.90–1.76)	0.174
Overweight 25 < = BMI < 30	0.85 (0.64–1.12)	0.246	0.94 (0.69–1.27)	0.665
Karnofsky Performance Status 50–80 (reference = 90–100)	1.28 (0.92–1.79)	0.139	1.24 (0.85–1.79)	0.26
Clinical Stage III (reference = II)	1.06 (0.83–1.35)	0.653	1.04 (0.80–1.36)	0.754
Intent to save sphincter (reference = no)	1.03 (0.78–1.35)	0.847	1.17 (0.87–1.58)	0.30

Dependent variable is GP5 Somewhat/Quite a bit/Very much vs. A little bit/Not at all

^a^ All covariates were entered simultaneously and are, therefore, mutually adjusted for in the multivariable model

^b^ Odds Ratio is expressed as 1-year increment

Table 4 shows associations between PRO or symptom scale scores, or single items from these scales, and post-chemotherapy/radiation GP5 stratified by whether the patient was treated with oxaliplatin or not. The associations were statistically significant for all tests except the following: FACT-C Social/Family Well-being for participants who did not receive oxaliplatin (*p* = 1.00). The probabilities that a one-unit increase in scale score or single item was association with worse GP5 side effect bother were generally similar between the oxaliplatin and non-oxaliplatin arms, and they were strongest for single items representing nausea and vomiting (FACT-C item GP2; SCL-17 vomiting item), single items related to abdominal pain (SCL-17 abdominal pain item), single items related to diarrhea and frequency of bowel movements (FACT-C item C5; SCL-17 diarrhea item; EORTC-CR38 item regarding frequency of bowel movements during the day), the EORTC-CR38 item regarding blood in stool, and FACT-NTX items regarding numbness and tingling in hands (NTX1) and feet (NTX2).

**Table 4:** Bivariate analyses of GP5 with patient-reported outcomes reflecting Health-Related Quality of Life and Symptomatic Side effects with overall side Bother Post- Chemotherapy/Radiation in patients treated with and without oxaliplatin (OXA)

PRO Scale	GP5 in patients treated with OXA		GP5 in patients treated without OXA	
	Probability (95% CI) ^a^	P-value ^†^	Probability (95% CI) ^a^	P-value ^†^
**FACT-C subscale scores** ^b^				
Physical Well-Being (0–28)	0.438 (0.431–0.445)	< 0.001	0.443 (0.437–0.448)	< 0.001
Social/Family Well-being (0–28)	0.491 (0.485–0.497)	0.075	0.497 (0.491–0.503)	1.00
Emotional Well-being (0–24)	0.478 (0.471–0.485)	< 0.001	0.476 (0.469–0.483)	< 0.001
Functional well-being (0–28)	0.473 (0.469–0.478)	< 0.001	0.48 (0.475–0.485)	< 0.001
Colorectal Cancer Scale (0–28)	0.466 (0.46–0.472)	< 0.001	0.473 (0.467–0.479)	< 0.001
**FACT-C individual items**				
C5: “I have diarrhea (diarrhoea)” ^c^	0.591 (0.571–0.612)	< 0.001	0.578 (0.557–0.599)	< 0.001
C3: “I have control of my bowels” ^b^	0.437 (0.414–0.46)	< 0.001	0.455 (0.433 -0.478)	0.002
GP2: “I have nausea” ^c^	0.607 (0.582–0.632)	< 0.001	0.628 (0.598–0.658)	< 0.001
**EORTC-CR38 subscale scores** ^c^				
GI Tract Subscale	0.508 (0.506–0.509)	< 0.001	0.507 (0.505–0.508)	< 0.001
Chemo Side Effects Subscale	0.506 (0.505–0.508)	< 0.001	0.506 (0.504–0.507)	< 0.001
Defecation Problems Subscale	0.508 (0.506–0.509)	< 0.001	0.506 (0.505–0.508)	< 0.001
**EORTC-CR38 items** ^c^				
Did frequent bowel movements occur during the day?	0.574 (0.542–0.605)	< 0.001	0.587 (0.554–0.619)	< 0.001
Did frequent bowel movements occur during the night?	0.557 (0.528–0.587)	0.004	0.572 (0.542–0.602)	< 0.001
Did you feel the urge to move your bowels without actually producing any stools?	0.575 (0.546–0.604)	< 0.001	0.567 (0.540–0.593)	< 0.001
Have you had blood in your stools?	0.584 (0.55–0.617)	< 0.001	0.577 (0.546–0.608)	< 0.001
**SCL-17 items** ^c^				
Bothered by: Diarrhea	0.579 (0.559–0.598)	< 0.001	0.582 (0.561–0.602)	< 0.001
Bothered by: Abdominal pain	0.596 (0.574–0.618)	< 0.001	0.586 (0.564–0.608)	0.014
Bothered by: Vomiting	0.610 (0.558–0.661)	0.001	0.627 (0.556–0.693)	0.040
Bothered by: Constipation	0.544 (0.52–0.567)	0.007	0.536 (0.514–0.558)	0.002
**FACT-NTX13 (0–52)** ^c^	0.520 (0.515–0.525)	< 0.001	0.519 (0.515–0.524)	< 0.001
**FACT-NTX13 items** ^c^				
NTX1: “I have numbness or tingling in my hands”	0.576 (0.551–0.601)	< 0.001	0.599 (0.562–0.634)	< 0.001
NTX2: “I have numbness or tingling in my feet”	0.576 (0.542–0.609)	< 0.001	0.567 (0.536–0.598)	< 0.001

(Dependent variable is GP5 as an ordinal variable; 0=“Not at all”, 1=“A little bit, 2=“Somewhat”, 4=“Quite a bit”, 5=“Very much”)

^a^ Values show the probability that participants with a one-unit increase in the HRQoL or symptom bother scale score or single item have more severe bother on GP5 than participants who do not have a one unit increase. A probability of 0.5 indicates no difference

^b^ Higher scores indicate better HRQoL or lower symptom bother

^c^ Higher scores indicate worse symptom bother

† P-values are calculated using the Wald statistic and adjusted for multiple tests using the Holm procedure

## Discussion

This study performed a post-hoc, secondary analysis of the R-04 trial to determine if overall side effect impact as captured by the GP5 item differed by treatment with or without oxaliplatin after post-neoadjuvant chemotherapy/radiotherapy. We found that receipt of oxaliplatin in combination with chemotherapy/radiation was associated with 1.58 times greater odds of reporting moderate to high overall side effect impact on the GP5 item compared to receiving chemotherapy/radiation alone.

The results of this study add to previous PRO analyses of the R-04 trial by examining the potential value of an overall side effect summary for patient reported treatment burden from different neoadjuvant treatment regimens for resectable rectal cancer. A previous secondary analysis of R-04 found higher Toxicity Index scores for treatment arms with oxaliplatin vs. those without oxaliplatin (*p* < 0.001) [10]. The Toxicity Index summarizes the entire toxicity experience rather than just the highest grade, typically using CTCAE but with recent adaptations for PROs [26]. However, a separate analysis of the PRO data from R-04 did not find differences between treatment arms in the FACT-C, SF-36 Vitality scale (fatigue), the SCL-17, the FACT-NTX Neurotoxicity Subscale scale and the FACT-NTX-4. One plausible explanation for why our results aligns well with the Toxicity Index results from R-04 but not with the previous PRO analyses in terms of distinguishing between treatment arms could be that the GP5 item captures cumulative toxicity burden of more than one toxicity. We point out the large effect of baseline GP5 on post-neoadjuvant chemotherapy/radiation. This finding has been observed previously [27], suggesting that a patient’s impression of their side effect bother is highly influenced by their starting levels of bother, even when this refers to bother with side effects from non-investigational treatments.

 A 2018 Food and Drug Administration (FDA)/Critical Path Institute workshop reported on the salience of the overall side effect impact concept over and above measurement of individual symptomatic side effects [11], and the relevance of GP5 for capturing overall side effect impact was further highlighted in the FDA’s Core Patient-Reported Outcomes in Cancer Clinical Trials guidance [2]. Independent measurement and comparison of a select number of symptomatic side effects may leave out important side effects and not address how patients weight each of the individual side effects on patients’ lives and, ultimately, the role of each of those side effects in an individual patient’s consideration of whether the treatment is ultimately tolerable [11]. Since R-04 participants receiving oxaliplatin had higher rates of high grade diarrhea, fatigue, and peripheral neuropathy, it is possible that GP5 was able to capture each patient’s individual experience with these multiple toxicities simultaneously. In addition, since it is only one item, administration of GP5 is brief and adds little to no burden to patients’ trial participation.

Consistent with our hypotheses, items and scales capturing diarrhea, vomiting, and neurotoxicity were most likely to be associated with higher side effect impact on GP5, even when rated on different HRQoL instruments. For example, association probabilities between GP5 and diarrhea items were similar for ratings from FACT-C (“I have diarrhea (diarrhoea)”), EORTC (frequent bowel movements occurring during day or not), and SCL-17 (Bother by: Diarrhea). In addition, the association probabilities between GP5 and FACT-NTX13 items on numbness and tingling in hands and feet were higher than they were for the overall FACT-NTX13 score, indicating that peripheral neuropathy associated with oxaliplatin was more bothersome than other side effects asked about by the FACT-NTX13 (e.g., discomfort in hands and feet, joint pain). This is consistent with the well-known severe peripheral neuropathy associated with oxaliplatin [28, 29]. Notably, GP5 was not associated with the EORTC-CR38 Chemotherapy Side Effects subscale. This is likely due to the items in this scale focusing on dry mouth, hair thinning, and changes in taste of food or drink, which were not commonly observed in any treatment arm [7]. Two of these items were re-worded in the shortened version of the EORTC-CR38, EORTC-CR29 [30]. Finally, we note that these associations were not limited to the oxaliplatin arms, and were also observed for the non-oxaliplatin arms. This stands to reason, since GP5 does not ask about oxaliplatin specifically and, therefore, should be sensitive to toxicities experience in any treatment arm. Though less common than in the oxaliplatin arms, patients in the non-oxaliplatin arms did experience appreciable rates of grade 3–5 diarrhea (6.9% among 5-FU and capecitabine without oxaliplatin), grade 3 nausea (1.3% among capecitabine without oxaliplatin), and grade 2–4 peripheral sensory neuropathy (2.2% among capecitabine without oxaliplatin) [7]. Similar to previous studies [27, 31], some patients reported some side effect bother at baseline. Previous analyses have found that patients may indicate side effect bother on GP5 at the baseline of a cancer trial prior to receipt of treatment due to expectancies about treatment or concomitant treatments not related to the investigational treatments [32].

We observed that women had worse overall side effect impact on the GP5 item, even in the multivariable logistic regression model that controlled for potential confounders. This finding aligns with those of Gresham, et al.’s analysis of R-04, which found that women had an increased probability of higher toxicity than men [10]. A body of previous research has established that women are more likely to experience adverse events associated with anti-cancer treatments, at least in part due to body composition and psychosocial factors that impact women differently than men [33, 34]. For example, a study by Lorusso and colleagues found that women reported some side effects, including vomiting, as being least tolerable more frequently than men [35]. More specifically, previous research has found greater sensitivity to toxicities of both oxaliplatin and capecitabine among women than men [36–38]. We note that we found no interaction between treatment arm and gender in this study, which may be explained by the higher likelihood of women having worse side effect bother regardless of treatment. More research is needed to understand whether women are more likely to report worse side effect bother across different cancer treatment settings.

Like any study, this study has important limitations to consider when interpreting its results. First, the analyses conducted here were secondary, as GP5 was not identified as an outcome in the trial protocol. Since the study was not necessarily designed for comparisons of patient-reported tolerability by treatment arm, it is possible that other design elements (e.g., more frequent GP5 assessment) would be better for testing differences in GP5 responses between treatments. Nonetheless, the current design allows for a unique opportunity to examine the isolated impact of oxaliplatin toxicities over and above standard of care chemotherapy for resectable rectal cancer. Second, despite the plausibility that GP5 differed between arms whereas HRQoL and symptom scales did not is due to effect of cumulative toxicity captured by GP5, this study could not test that explanation directly. Future studies should seek to examine this link more directly. Finally, the results presented here could be sensitive to different ways of operationalizing GP5 as an outcome. A recent study advanced suggestions for how best to operationalize GP5 as an outcome in cancer trials, which included the approach taken in the current study [dichotomizing GP5 response as moderate to high bother (“Somewhat”/“Quite a bit”/“Very much” vs. low bother (“Not at all”/“A little bit”)] [39], but future studies should examine other ways to use GP5 in comparisons of treatment arms.

In conclusion, this study leveraged one of the largest clinical trials examining neoadjuvant treatment approaches in resectable rectal cancer to test a novel approach to understanding and comparing treatment tolerability using patient report. This study’s results reiterate that oxaliplatin in combination with standard of care chemotherapy and radiotherapy is less tolerable to patients than standard of care alone. The decreased tolerability of oxaliplatin needs to be considered in the context of its failure to demonstrate superiority in tumor control, disease-free survival, and overall survival. Furthermore, our findings demonstrate the relevance of an overall side effect impact item for investigating cancer treatment tolerability, particularly between arms of varying side effects.

## Electronic supplementary material

Below is the link to the electronic supplementary material.

Supplementary Material 1

## Data Availability

Data can be requested through the NCTN/NCORP data archive (https://nctn-data-archive.nci.nih.gov).
