# Supplementary figures and images for: Overall side effect assessment of oxaliplatin toxicity in rectal cancer patients in NRG oncology/NSABP R04
Source: Qual Life Res. 2024 Jul 30;33(11):3069–79. doi: 10.1007/s11136-024-03746-5 (PMC11541265; doi:10.1007/s11136-024-03746-5)

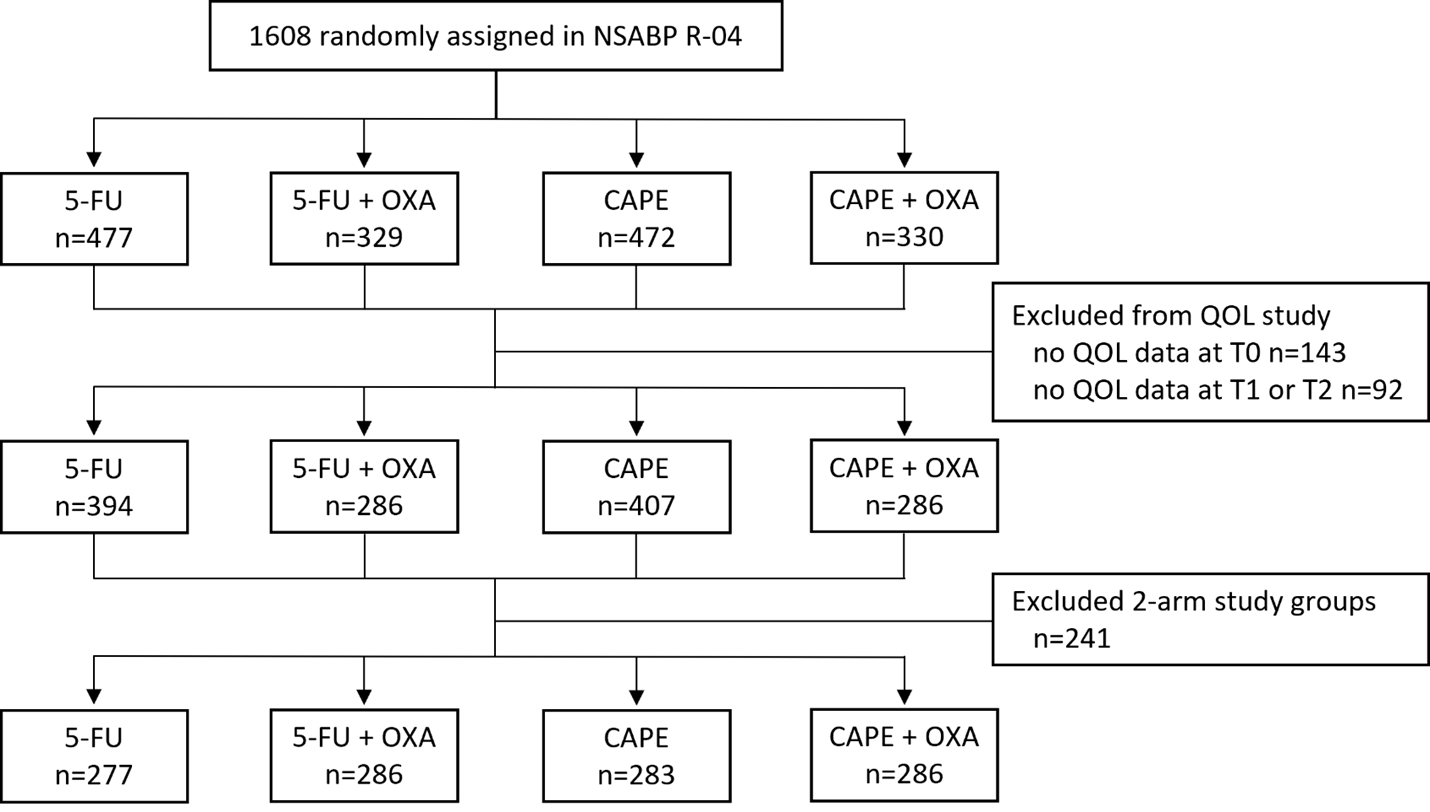

Supplement: Supplementary file 1 — Supplementary Material 1 [file 11136_2024_3746_MOESM1_ESM.png]
